# Sulfatide-Hsp70 Interaction Promotes Hsp70 Clustering and Stabilizes Binding to Unfolded Protein

**DOI:** 10.3390/biom5020958

**Published:** 2015-05-15

**Authors:** Yoichiro Harada, Chihiro Sato, Ken Kitajima

**Affiliations:** 1 The Laboratory of Animal Cell Function, Bioscience and Biotechnology Center, Nagoya University, Nagoya 464-8601, Japan; E-Mails: yoharada@m.kufm.kagoshima-u.ac.jp (Y.H.); chi@agr.nagoya-u.ac.jp (C.S.); 2 Graduate School of Bioagricultural Sciences, Nagoya University, Nagoya 464-8601, Japan

**Keywords:** high molecular-weight complex, Hsp70, oligomerization, sulfatide

## Abstract

The 70-kDa heat shock protein (Hsp70), one of the major stress-inducible molecular chaperones, is localized not only in the cytosol, but also in extracellular milieu in mammals. Hsp70 interacts with various cell surface glycolipids including sulfatide (3'-sulfogalactosphingolipid). However, the molecular mechanism, as well as the biological relevance, underlying the glycolipid-Hsp70 interaction is unknown. Here we report that sulfatide promotes Hsp70 oligomerization through the N-terminal ATPase domain, which stabilizes the binding of Hsp70 to unfolded protein *in vitro*. We find that the Hsp70 oligomer has apparent molecular masses ranging from 440 kDa to greater than 669 kDa. The C-terminal peptide-binding domain is dispensable for the sulfatide-induced oligomer formation. The oligomer formation is impaired in the presence of ATP, while the Hsp70 oligomer, once formed, is unable to bind to ATP. These results suggest that sulfatide locks Hsp70 in a high-affinity state to unfolded proteins by clustering the peptide-binding domain and blocking the binding to ATP that induces the dissociation of Hsp70 from protein substrates.

## 1. Introduction

Molecular chaperones constitute the central core of a protein quality control system. When unfolded proteins are generated, molecular chaperones bind to them, thereby preventing their non-functional, cytotoxic aggregate formation [1]. In the cytosol of mammalian cells, the stress-inducible 70-kDa heat shock protein (Hsp70) is one of the major molecular chaperones that are highly expressed under stress conditions [2,3]. Hsp70 has an intrinsic ATPase activity that regulates association and dissociation between Hsp70 and protein substrates [4,5,6,7,8,9]. In the ATP-bound state, Hsp70 shows low affinity for protein substrates. When Hsp70 hydrolyzes ATP to ADP, the protein substrates are tightly bound to Hsp70.

Besides the cytosolic localization of Hsp70, this molecular chaperone is also known to be released from various types of cells into the extracellular space by necrosis [10], exosome-mediated trafficking [11,12], and unconventional secretion pathway [13]. Moreover, Hsp70 is expressed on the surface of cancer cells by unclarified mechanisms that involve lipid rafts [14,15]. Although precise functions of the extracellular Hsp70 have not yet been fully understood, evidence is accumulating that cell surface Hsp70s are involved in cell-cell recognitions [16,17]. It has also been reported that exogenously administered Hsp70 enhances the transfer of antigens to dendritic cells, cross-presentation [18,19], and cytotoxic T lymphocyte (CTL) responses against antigenic peptides [18].

Hsp70 possesses a unique property to bind to various lipids, such as fatty acids [20], phospholipids [21,22], and glycolipids [23,24,25]. Sulfatide (3'-sulfogalactosphingolipid) is one of the well-characterized glycolipids that serve as an Hsp70 receptor presented on the surface of pro- and eukaryotic cells [16,23,26,27,28,29,30,31]. As an extension of these previous studies, we have demonstrated that sulfatide-Hsp70 interaction induces the formation of characteristic high molecular-weight (HMW) complexes of Hsp70 (HMW Hsp70) [24]. However, the molecular mechanism, as well as the biological relevance, of the HMW complex formation is unknown. In the present study, we attempted to gain mechanistic insights into the lipid-regulated Hsp70 functions by characterizing the sulfatide-Hsp70 HMW complex *in vitro*.

## 2. Results

### 2.1. Characterization of the Purified Hsp70 and Sulfatide by Gel-Filtration Chromatography

**Figure 1:**
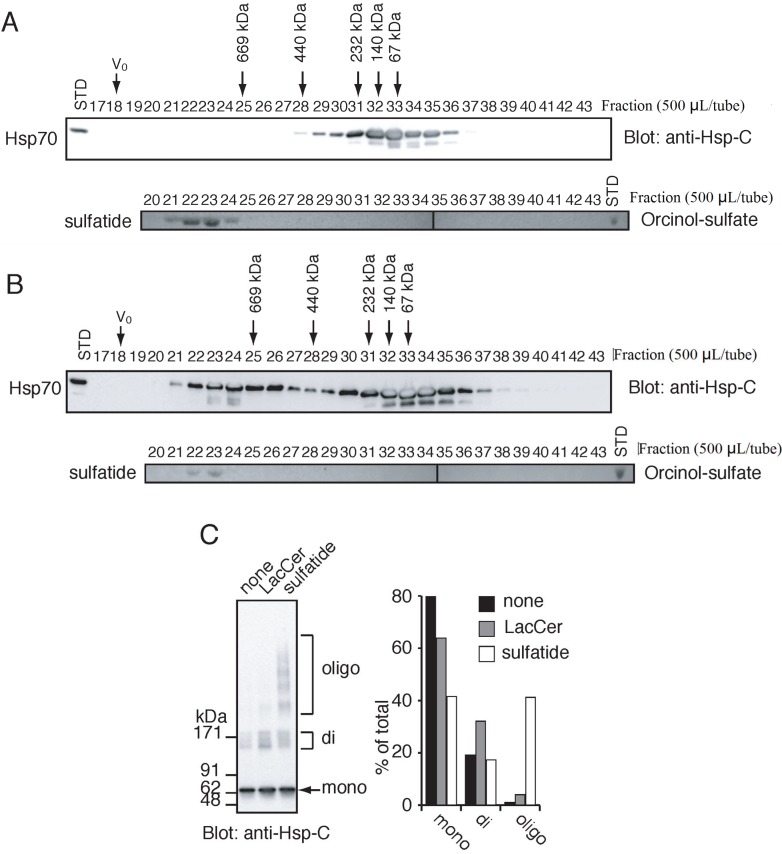
Sulfatide-Hsp70 interaction induces oligomerization of 70-kDa heat shock protein (Hsp70). (**A**) Hsp70 (top panel) and sulfatide (bottom panel) were separately analyzed by gel filtration chromatography. Fractions and external standards (STD; Hsp70, 35 ng; sulfatide, 2 μg) were analyzed by Western blot using anti-Hsp-C antibody for Hsp70 or TLC/orcinol sulfate method for sulfatide. V_0_ indicates void volume. Thyroglobulin (669 kDa), Ferritin (440 kDa), catalase (232 kDa), aldolase (140 kDa) and albumin (67 kDa) were used as molecular weight markers; (**B**) Hsp70 and sulfatide were co-incubated and analyzed as in **A**; (**C**) Hsp70 was incubated with or without lactosylceramide (LacCer) and sulfatide and chemically cross-linked with glutaraldehyde. The products were separated by SDS-PAGE (stacking gel, 3% and separating gel, 3%–7%) and analyzed by Western blot using anti-Hsp-C antibody (left panel). Relative amounts of mono-, di-, and oligomers of Hsp70 were quantitated (right panel); (**D**) The Hsp70-sulfatide complex was analyzed by native-PAGE, followed by Western blot using anti-Hsp-C antibody. Monomer and dimer indicate the mono- and dimeric form of Hsp70.

We previously demonstrated that sulfatide induces the formation of the HMW Hsp70 based on the results of native-PAGE analysis on Hsp70-lipid interaction [24]. However, the migration of compounds in native PAGE is affected by both charges and molecular masses, which made it difficult to estimate native molecular masses of the HMW Hsp70. We thus employed gel-filtration chromatography to simply obtain size information. Under a physiological extracellular salt condition (150 mM NaCl), the purified Hsp70 was eluted between tubes 28–37 (up to 440 kDa) with a peak top at around molecular masses of 67–140 kDa (Figure 1A, top panel), showing that Hsp70 mainly exists as mono- to dimer, and a small fraction of Hsp70 forms higher ordered oligomers. The oligomer formation is most likely because of a self-oligomerization property of Hsp70 [32,33]. Sulfatide was detected at fractions with molecular masses of >669 kDa (Figure 1A, bottom panel), revealing that sulfatide forms micelles in aqueous solutions. As an average size of sulfatide micelles is approximately 900 kDa, >745 molecules of sulfatide were estimated to form a micelle.

### 2.2. Sulfatide Induces Higher Ordered Oligomerization of Hsp70

Next, we determined the molecular mass of the sulfatide-induced HMW Hsp70. Upon incubation with sulfatide, the apparent molecular mass of Hsp70 was substantially increased to sizes ranging from 440 kDa to >669 kDa (Figure 1B, top panel). Although some Hsp70 co-migrated with sulfatide micelles (Figure 1B, compare top and bottom panels), they were not always associated with each other, as the sulfatide micelle-unbound form of Hsp70 was also detected in high molecular weight regions (Figure 1B, fractions 25–27). This result led us to speculate that sulfatide promotes the formation of higher ordered Hsp70 oligomers. To test this hypothesis, Hsp70 was cross-linked with glutaraldehyde in the presence or absence of sulfatide and analyzed by SDS-PAGE. Chemical cross-linking resulted in the formation of ladder bands that were reactive with anti-Hsp70 antibody (Figure 1C). Based on their molecular masses, it was suggested that in the absence of sulfatide, >90% of Hsp70 formed mono- and dimer, and little larger oligomers were observed. However, incubation with sulfatide increased the relative amounts of the higher ordered Hsp70 oligomers. Lactosylceramide, which has been shown to have no ability to induce HMW complex formation of Hsp70 [24], did not alter the oligomeric state of Hsp70. Collectively, this result indicated that sulfatide-Hsp70 interaction induces the higher ordered oligomerization of Hsp70.

### 2.3. Sulfatide-Induced Oligomerization of Hsp70 through the ATPase Domain

**Figure 2:**
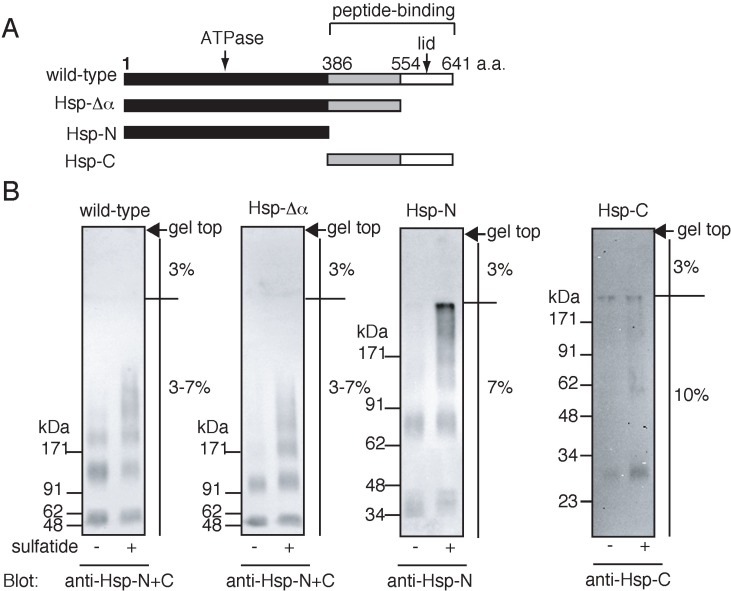
The ATPase domain of Hsp70 mediates sulfatide-induced oligomerization of Hsp70. (**A**) Schematic representation of wild-type and truncation mutants of Hsp70. Hsp70-Δα, Hsp70-N and Hsp-C lack the lid domain, the peptide-binding domain and the ATPase domain, respectively. The number of amino acid residues (a.a.) were indicated on the scheme; (**B**) Hsp70 was incubated with (+) or without (−) sulfatide, cross-linked with glutaraldehyde, and analyzed by Western blot using anti-Hsp-N and anti-Hsp-C antibodies.

Next, we tested whether the ATPase domain is involved in sulfatide-induced oligomerization of Hsp70, as this domain has been implicated in the sulfatide-induced formation of the HMW Hsp70 in native-PAGE [24]. When the ATPase domain (Figure 2A) was incubated with sulfatide and chemically cross-linked, higher ordered oligomers with molecular masses >91.3 kDa, in addition to the mono- and dimers, were detected (Figure 2B). However, the ATPase domain formed only mono- and dimers in the absence of sulfatide. It has been reported that the lid domain of the peptide-binding domain (Figure 2A) plays crucial roles in spontaneous self-oligomerization of Hsp70 [32]. However, its truncation did not affect sulfatide-induced oligomerization of the mutant Hsp70 (Figure 2B). Moreover, peptide-binding domain (Figure 2A) did not form the sulfatide-induced oligomers (Figure 2B). All these data indicated that the ATPase domain is sufficient for the sulfatide-induced oligomerization of Hsp70.

### 2.4. Effects of K^+^, Na^+^ and Nucleotides on the Sulfatide-Induced Formation of the HMW Hsp70

**Figure 3:**
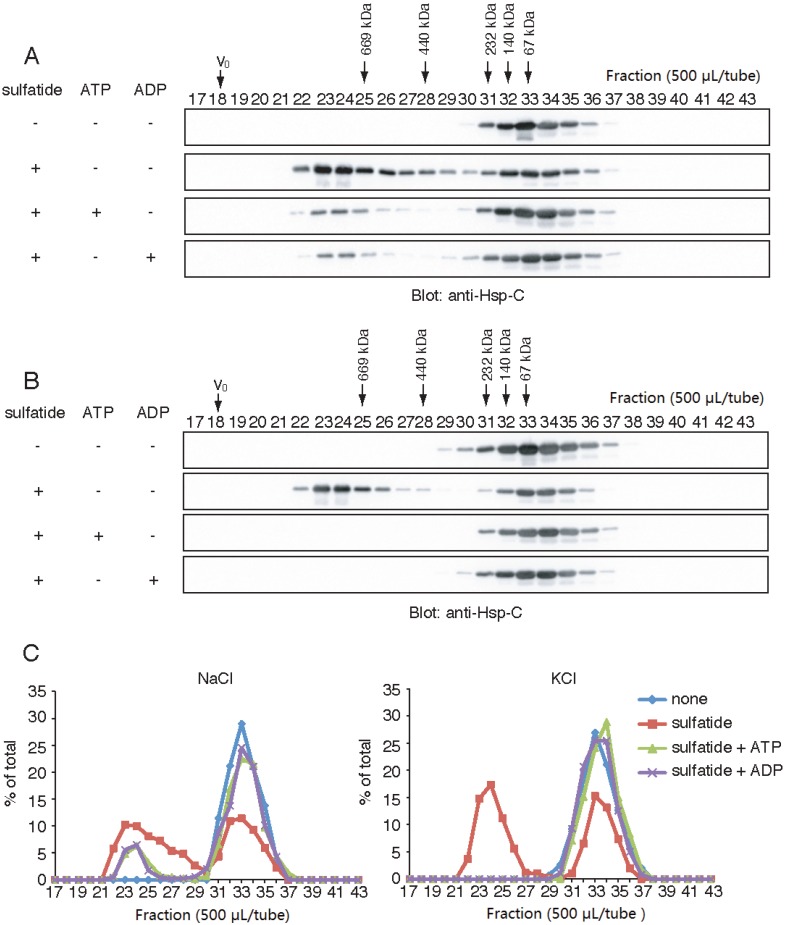
ATP and ADP inhibits the sulfatide-induced formation of the high molecular-weight (HMW) Hsp70. Wild-type Hsp70 was incubated with (+) or without (−) sulfatide, ATP and ADP in a buffer containing either 150 mM NaCl (**A**) or KCl (**B**), and separated by gel-filtration chromatography under the same salt conditions. Fractions (500 μL) were precipitated with trichloroacetic acid (TCA) and analyzed by Western blot using anti-Hsp-C antibody. V_0_ indicates void volume. Thyroglobulin (669 kDa), Ferritin (440 kDa), catalase (232 kDa), aldolase (140 kDa) and albumin (67 kDa) were used as molecular weight markers; (**C**) Quantification of **B**. Total intensity of Hsp70 was set to 100%.

Hsp70 functions are regulated by nucleotide-binding states. Since it has been shown that ATP inhibits spontaneous self-oligomerization of cognate Hsp70 irrespective of the presence of Na^+^ or K^+^ [34], we examined the effects of ATP on the sulfatide-induced formation of the HMW Hsp70. Hsp70 was co-incubated with sulfatide and ATP in the presence of 150 mM NaCl, and separated by gel-filtration chromatography under the same salt condition. In the presence of 1 mM ATP, the sulfatide-induced formation of the HMW Hsp70 was only partially inhibited (Figure 3A). We found that 1 mM ADP shows a similar partial inhibitory effect to ATP. Interestingly, when NaCl was substituted to the same concentration of KCl (Figure 3B), these nucleotides were able to completely inhibit the sulfatide-induced formation of HMW Hsp70. Collectively, these results indicated that ATP and ADP impair the sulfatide-induced formation of the HMW Hsp70 and the inhibitory effects are pronounced in the presence of 150 mM KCl.

### 2.5. Loss of ATP-Binding Activity of the HMW Hsp70

**Figure 4:**
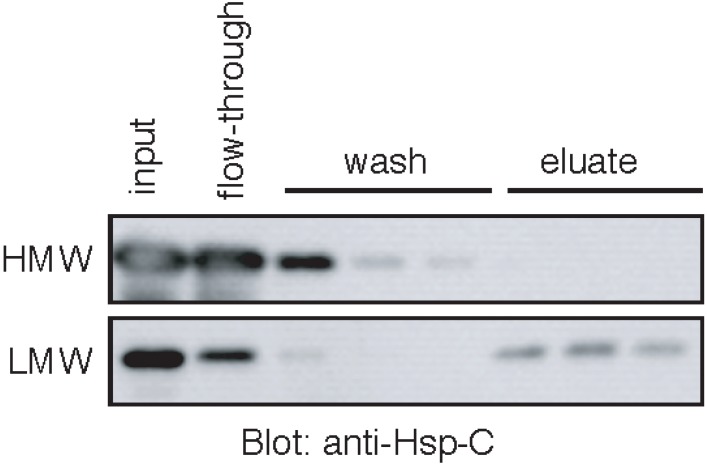
The HMW Hsp70 loses ATP-binding activity. The HMW (top panel) and low molecular weight (LMW) (bottom panel) Hsp70 (1 mL each) prepared as described in “Experimental Section” were incubated with ATP-agarose. The beads were washed and the bound Hsp70 was eluted with 5 mM ATP. All fractions (100 μL) were precipitated with TCA and analyzed by Western blot using anti-Hsp-C antibody.

Next, we tested whether the HMW Hsp70 possesses ATP-binding activity. The HMW Hsp70 and the low molecular weight (LMW) Hsp70 were prepared by gel-filtration chromatography of an incubation of Hsp70 and sulfatide, and these fractions were incubated with ATP-immobilized beads. Most of the HMW Hsp70 was detected in flow-through and wash fractions (Figure 4, top panel), whereas LMW Hsp70, if not all, was bound to the ATP-immobilized beads (Figure 4, bottom panel). This result suggested that once formed, the HMW Hsp70 loses ATP-binding activity.

### 2.6. Stable Complex Formation between the HMW Hsp70 and Unfolded Protein

**Figure 5:**
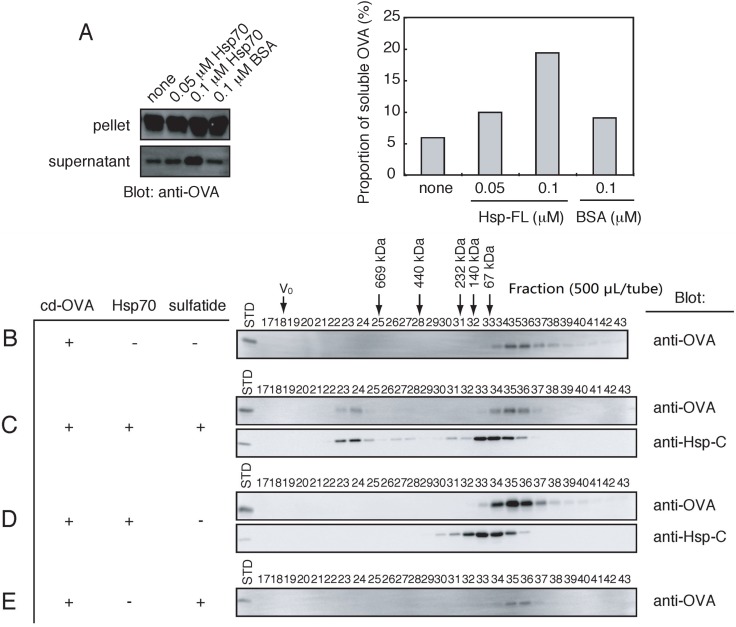
Sulfatide-induced formation of the HMW Hsp70 stabilizes the binding between Hsp70 and chemically denatured ovalbumin (cd-OVA). (**A**) Hsp70 or bovine serum albumin (BSA) was incubated with cd-OVA for 30 min at 42 °C. After centrifugation, the supernatant (sup.) and the pellet (ppt.) fractions were analyzed by SDS-PAGE, followed by Western blot using anti-OVA antibody (left panel). Total intensity of cd-OVA was set to 100% and the relative amounts of cd-OVA in the supernatant fraction (soluble OVA) were indicated (right panel); (**B**–**E**) Hsp70, sulfatide, and cd-OVA were incubated as indicated combinations and separated by gel-filtration chromatography. Fractions (500 μL) precipitated with TCA and external standards (STD; Hsp70, 35 ng; cd-OVA, 10 ng) were analyzed by Western blot using anti-Hsp-C and anti-OVA antibodies. V_0_ indicates void volume. Thyroglobulin (669 kDa), Ferritin (440 kDa), catalase (232 kDa), aldolase (140 kDa) and albumin (67 kDa) were used as molecular weight markers.

To test whether Hsp70-sulfatide interaction affects the protein substrate-binding activity of Hsp70, chemically denatured ovalbumin (cd-OVA), whose peptide fragments can be bound to Hsp70 [35], was used as a model protein. First, we tested whether Hsp70 prevents cd-OVA from the formation of heat-induced aggregation. As shown in Figure 5A, the relative amounts of the soluble form of cd-OVA were increased in the presence of Hsp70, whereas bovine serum albumin (BSA) had no effects on the solubility of cd-OVA. This result indicated that Hsp70 has the chaperoning activity for cd-OVA. Next, using gel-filtration chromatography, we tested whether the interaction between Hsp70 and cd-OVA is modulated by sulfatide. The cd-OVA alone was eluted in positions immediately after 67 kDa (Figure 5B), which is in good agreement with the predicted molecular size of OVA (45 kDa). When incubated with Hsp70 and then sulfatide, cd-OVA was detected in HMW regions where Hsp70 was co-eluted (Figure 5C, tubes 23–28). However, the co-elution between Hsp70 and cd-OVA was not observed in LMW regions (Figure 5C, tubes 30–36). Similarly, in the absence of sulfatide, no apparent association between Hsp70 and cd-OVA was observed (Figure 5D). Sulfatide micelles did not alter the elution pattern of cd-OVA (Figure 5E). All these results indicate that sulfatide strengthens the association between Hsp70 and cd-OVA by clustering Hsp70.

## 3. Discussion

In the present study, we demonstrated that Hsp70-sulfatide interaction induces Hsp70 oligomerization via a novel mechanism involving the N-terminal ATPase domain. The sulfatide-induced clustering of Hsp70 stabilized the interaction between Hsp70 and an unfolded protein. These effector functions of sulfatide may improve the antigen-transfer activity of Hsp70 [18,19] by concentrating antigens on the Hsp70 cluster.

Gel-filtration chromatographic studies on the Hsp70-sulfatide interaction revealed that the sulfatide-induced formation of the HMW Hsp70 is not due to a permanent association of Hsp70 to sulfatide micelles, as not all of the HMW Hsp70 was co-eluted with the micelles. This finding is consistent with the fact that Hsp70 formed higher ordered oligomers in the presence of sulfatide. Our data showed that the complex formation of Hsp70 requires excess amounts of sulfatide. Although why such high concentrations of sulfatide are required to induce the formation of HMW complex of Hsp70 remains to be clarified, it has been shown that the binding of bovine Hsc70 and sulfatide is inhibited by a water-soluble form of sulfatide analog at concentrations ranging from 100–300 μM [31].

Our data clearly indicate that in aqueous solutions, sulfatide forms micelles and induces the formation of the HMW complex of Hsp70, revealing that Hsp70 interacts with sulfatide micelles. By contrast, it has been reported that Hsp70 does not form a stable complex with sulfatide-containing liposomes [25]. Since a fraction of the HMW complex of Hsp70 is dissociated from sulfatide micelles, it is speculated that Hsp70-sulfatide interaction may result in the extraction of sulfatide from micelles or liposomes. However, whether Hsp70 interacts with sulfatide presented on the surface of lipid bilayer is currently unknown. Regarding this issue, it is known that glycosphingolipids including sulfatide are enriched in lipid rafts [36,37], raising the possibility that such membrane environment may provide the site for Hsp70 to interact with sulfatide. This attractive hypothesis, however, needs to be validated in future studies.

It has been reported that Hsp70 spontaneously forms self-oligomers through the peptide-binding domain [32]. However, this domain was dispensable for the sulfatide-induced self-oligomerization and the ATPase domain is sufficient for this process. This observation further indicates that the sulfatide-induced formation of HMW complex of Hsp70 results from the direct binding of the ATPase domain to sulfatide [24,29]. Upon binding to sulfatide, the ATPase domain may change its conformation, resulting in the exposure of unidentified oligomeric sites. Structural flexibility of the ATPase domain is likely, because it has been reported that the ATPase domain of hexokinase, which shows a high structural similarity to that of Hsp70 [4,38], undergoes conformational changes upon binding to glucose, leading to the formation of the catalytically active ATP-binding pocket [39,40].

Our analysis showed that the sulfatide-induced formation of HMW complex of Hsp70 is not due to the non-specific denaturation of proteins, as the peptide-binding domain of Hsp70 and cd-OVA alone did not form aggregates in the presence of sulfatide. However, the issue of whether the formation of the HMW complex of Hsp70 is reversible is currently unknown. It has been shown that ATP and substrate peptide reverses the spontaneous oligomerization of Hsc70 [34]. However, our data showed that the sulfatide-induced HMW complex of Hsp70 lost ATP-binding activity, implying that ATP does not induce the dissociation of the HMW complex of Hsp70. Furthermore, the incubation with cd-OVA did not induce the dissociation of the HMW complex of Hsp70, indicating that the interaction between Hsp70 and substrate peptides is not sufficient to dissociate the HMW complex. The identification of mechanisms underlying the reversibility of the HMW complex of Hsp70 may provide insights into important aspect of the release of substrate peptides from Hsp70.

The sulfatide-induced formation of the HMW Hsp70 was completely abolished by ATP and ADP in the presence of 150 mM KCl, and the inhibitory effect was attenuated by substitution of KCl to NaCl. Crystal structures of the ATPase domain of cognate Hsp70 in complex with ADP and Pi revealed that Hsp70 has two monovalent cation-binding sites that can accommodate both K^+^ and Na^+^ ions [41]. These cations influence the position of the nucleotide in the catalytic pocket [41], and K^+^ ion is a preferred co-factor for ATP hydrolysis [42]. These experimental findings indicated that the Hsp70-nucleotide binding with the proper orientation inhibits the sulfatide-induced formation of the HMW Hsp70. Intracellular environment contains high concentrations of K^+^ ion and ~5 mM ATP [43], implying that the sulfatide-regulated oligomerization of Hsp70, if any, is suppressed inside of cells. In contrast, Na^+^ ion is predominant in the extracellular space where sulfatide is exposed. Therefore, we speculate that the sulfatide-induced formation of the HMW Hsp70 may occur outside of cells.

Our results showed that the sulfatide-induced HMW Hsp70 strongly binds to cd-OVA. In contrast, LMW Hsp70 did not form a stable complex with cd-OVA. Thus, sulfatide stabilizes association between Hsp70 and cd-OVA by promoting Hsp70 oligomerization. Because the peptide-binding domain is not essential for the oligomerization, it is reasonable to assume that a tight interaction between Hsp70 and cd-OVA is achieved by clustering the peptide-binding domain. We also showed that the HMW Hsp70 is unable to bind to ATP. This is most likely due to inaccessibility of ATP to the binding site by steric hindrance caused by Hsp70 oligomerization, as Hsp70 has a putative sulfatide-binding pocket adjacent, but not identical, to the ATP-binding site [29,31]. The blockade of ATP-binding may prevent the HMW Hsp70-substrate complex from ATP-induced dissociation.

In conclusion, our data showed that sulfatide can act as a modulator for Hsp70 functions. The modulatory effects include the clustering of Hsp70, the downregulation of ATP-binding activity and the stabilization of the Hsp70-substrate interaction. Our findings also suggested that extracellular environment may potentiate these effects. We have previously identified several glycoconjugates, such as gangliosides and glycosaminoglycans (GAGs), as Hsp70 interactors [24,44]. Notably, the interaction between Hsp70 and an artificial heparin dimer does not seem to induce Hsp70 oligomerization [44], indicating that modulatory effects may be dependent on glycoconjugates. Further studies will be needed to clarify how glycoconjugates influence Hsp70 functions.

## 4. Experimental Section

### 4.1. Materials

Sulfatide, lactosylceramide (LacCer), ovalbumin (OVA), anti-OVA, peroxidase-conjugated goat antibody against rabbit IgG and ATP-agarose were purchased from Sigma-Aldrich (St. Louis, MO, USA). Peroxidase-conjugated goat antibody against rat IgG was from American Qualex (San Clemente, CA, USA). Sephacryl S-300 was from GE healthcare (Buckinghamshire, UK). Anti-Hsp-N and anti-Hsp-C were prepared as described [24].

### 4.2. Protein Purification

Recombinant mouse Hsp70, Hsp-N and Hsp-C were prepared as described [24]. Hsp-Δα that lacks the C-terminal lid domain (amino acids 555 to 641) of Hsp70 was prepared as follows. Hsp-Δα was amplified by polymerase chain reaction (PCR) with sense and antisense primers, 5'-CGGGATCCATGGCCAAGAACACGGCGAT-3' and 5'-CCCAAGCTTCTACTCCACGGCGCTCTTCA-3' (*Bam*H I and *Hin*d III sites are underlined). The amplified DNA was cloned into pET32a (+) vector through the *Bam*H I and *Hin*d III sites (pHsp-Δα). DNA sequence was determined by deoxynucleotide chain termination method [45]. BL21 (DE3) pLysS cells were transformed with pHsp-Δα encoding thioredoxin (Trx)-fused Hsp-Δα and the expressed Trx-fused Hsp-Δα were captured from cell lysate on a Ni-NTA-Agarose column (QIAGEN). The column was washed with phosphate buffered saline (137 mM NaCl, 8.1 mM Na_2_HPO_4_, 2.68 mM KCl and 1.47 mM KH_2_PO_4_, pH7.4), and with 20 mM Tris-HCl, pH 7.5 (TB), 50 mM NaCl (TBS) containing 10 mM imidazol. Trx-fused proteins were eluted with TBS containing 500 mM imidazol. After changing the buffer to TBS by ultrafiltration using Amicon Ultra-15 (Merck Millipore, Billerica, MA, USA), Trx-fused Hsp-Δα was digested with recombinant enterokinase (Merck Millipore, Billerica, MA, USA) in the presence of 1 mM CaCl_2_. After adjusting to 25 mM magnesium acetate, the digest was applied onto ATP-Agarose column equilibrated with TB containing 50 mM KCl and 25 mM magnesium acetate. The column was washed with six column volumes of TB containing 500 mM KCl and 25 mM magnesium acetate, eluted with six column volumes of TB containing 50 mM KCl and 25 mM magnesium acetate, and then eluted with six column volumes of TB containing 50 mM KCl, 25 mM magnesium acetate, and 5 mM ATP to obtain the Trx-fused Hsp-Δα. The eluate was loaded onto a Ni-NTA column, and the flow through fraction was subjected to Q-Sepharose chromatography. After washing with ten column volumes of TB, Hsp-Δα was eluted with TB containing 75 mM NaCl. The Hsp-Δα was dialyzed once against PBS containing 25 mM KCl, 10 mM ammonium sulfate, 0.1 mM dithiothreitol and 0.1 mM EDTA, and twice against PBS, and concentrated. Protein concentration was estimated by the intensity of the staining with Coomassie Brilliant Blue (CBB) on SDS-polyacrylamide gel electrophoresis (PAGE) using bovine serum albumin (BSA) as a standard.

### 4.3. Gel-Filtration Chromatography 

For Figure 1, Hsp70 (0.1 μM) was incubated with or without sulfatide (250 μM) in PBS (1.0 mL) at 4 °C for 1.5 h, followed by further incubating at 30 °C for 10 min. The reaction mixture was subjected to Sephacryl S-300 chromatography (ϕ 1.5 × 59.0 cm) equilibrated with PBS and the eluates (500 μL/fraction) were collected. Four hundred microliters of each fraction was incubated with 3% trichloroacetic acid (TCA) at room temperature for 10 min and centrifuged at 20,000× *g* for 5 min. The precipitates were analyzed by SDS-PAGE (stacking gel, 3%; separating gel, 10%) followed by Western blotting using anti-Hsp-C antibody (1:5000 dilution). Sulfatide was detected by thin layer chromatography (TLC)/orcinol sulfate method [46]. For Figure 1A,B, 400 μL and 100 μL of fractions were spotted onto TLC plates. The plates were developed by chloroform/methanol/water (55:45:10, vol/vol/vol).

For Figure 3, Hsp70 and sulfatide were incubated with or without 1 mM ATP or ADP, and analyzed by Sephacryl S-300 essentially the same as described above, except that a buffer containing 25 mM Hepes-KOH, pH7.4 and either 150 mM KCl or 150 mM NaCl was used instead of PBS.

### 4.4. Cross-Linking Experiments

Hsp70, Hsp-Δα, Hsp-N and Hsp-C (0.1 μM each) was incubated with or without 250 μM sulfatide in PBS (50 μL) at 4 °C for 1.5 h. The reaction mixtures were cross-linked with glutaraldehyde (0.1% at final concentration) for 10 min at 30 °C. To terminate cross-linking, the reaction mixtures were incubated with Tris-HCl, pH7.5 (0.1 M at final concentration) for 5 min at 0 °C, followed by incubating with 3% trichloroacetic acid for 10 min at room temperature. After centrifugation at 20,000× *g* for 5 min, the precipitates were analyzed by SDS-PAGE (stacking gel, 3%; separating gel, 3%–7% for Hsp70 and Hsp-Δα, 7% for Hsp-N), followed by Western blotting using anti-Hsp-N (1:500 dilution) and/or anti-Hsp-C (1:2500 dilution) antibodies.

### 4.5. ATP-Agarose Binding Assay

Hsp70 (0.1 μM) and sulfatide (250 μM) in PBS (1.0 mL) was incubated at 4 °C for 1.5 h, followed by further incubation at 30 °C for 10 min. After Sephacryl S-300 column (ϕ 1.5 × 59.0 cm) chromatography, the eluates were pooled into two fractions, fraction I (fractions 22–25; HMW) and fraction II (fractions 31–37; LMW). Each fraction (1.0 mL) that was adjusted to 25 mM magnesium acetate was incubated with ATP-Agarose beads (100 μL) equilibrated with binding buffer (PBS containing 25 mM magnesium acetate) at 4 °C for 15 min with gentle shaking. After washing the beads three times with the binding buffer (100 μL each), the bound protein was sequentially eluted three times with 5 mM ATP in the binding buffer (100 μL each). Each fraction was precipitated with the trichloroacetic acid treatment as described above and subjected to SDS-PAGE (stacking gel, 3%; separating gel, 10%), followed by Western blotting using anti-Hsp-C antibody (1:5000 dilution).

### 4.6. Interaction between Hsp70 and cd-OVA

OVA (4.4 μM) was denatured in 32 mM Hepes-KOH, pH8.0, containing 6.0 M guanidine-HCl, 1 mM EDTA, and 50 mM KCl at 37 °C for 30 min. To analyze the chaperoning activity of Hsp70, chemically denatured OVA (cd-OVA; 44 nM) thus obtained was incubated with Hsp70 (0.05 and 0.1 μM) or bovine serum albumin (BSA; 0.1 μM) for 30 min at 42 °C. After centrifugation at 15,000× *g* for 15 min, the pellet was resuspended in the equal volume of the supernatant and analyzed by SDS-PAGE, followed by Western blot using anti-OVA antibody (1:3000 dilution). For the analysis of the interaction between Hsp70 and cd-OVA by gel-filtration chromatography, cd-OVA was diluted 100-fold into the Hsp70 (0.1 μM), and incubated at 30 °C for 10 min. Alternatively, 10 μL of cd-OVA (88 nM) was diluted 50-fold into 490 μL of Hsp70 (0.2 μM) and incubated at 30 °C for 10 min, followed by addition of 500 μL of sulfatide (final 250 μM). Immediately after final incubation at 30 °C for 10 min, the mixture was subjected to Sephacryl S-300 chromatography (ϕ 1.5 × 59.5 cm) as described in “Gel-filtration chromatography”. Each fraction (400 μL) was precipitated with trichloroacetic acid (3%), boiled in Laemmli buffer at 100 °C for 3 min and separated by SDS-PAGE (stacking gel, 3%; separating gel, 10%), followed by Western blotting using anti-OVA (1:3000 dilution) or anti-Hsp-C (1:5000 dilution) antibody.

## 5. Conclusions

In the present study, we found that the binding of sulfatide to Hsp70 induces the formation of HMW complex of Hsp70. The complex formation is mediated through the ATPase domain and the peptide-binding domain is dispensable for this process. The sulfatide-induced formation of the HMW Hsp70 is only partially inhibited by ATP and ADP in the presence of physiological concentrations of NaCl. Once the HMW Hsp70 is formed, the complex completely loses the binding activity to ATP-agarose, while the HMW Hsp70 tightly binds to cd-OVA. Although our results clearly indicated that sulfatide acts as a modulator for the functions of Hsp70 *in vitro*, which may improve antigen-transfer activity of Hsp70, major questions remained to be addressed include whether the HMW Hsp70 is formed by sulfatide *in vivo* and what could be the biological functions of the complex. We have shown that acidic phospholipids, gangliosides and glycosaminoglycans also serve as receptors for Hsp70. Much more studies will be needed to clarify the potential roles of these compounds in Hsp70 biology.
